# The strength and weakness of Mongolian healthcare: from nomadic Gobi to Ulaanbaatar

**DOI:** 10.7189/jogh.15.03015

**Published:** 2025-03-14

**Authors:** Wei-Ti Chen, Otgonchimeg Mangal, Khulan Munkhbaatar, Enkhtuya Vankhuu, Rachel HA Arbing, Yae Yoshino, 2024 Sophia University Global Health Practicum Team, Riko Hirata, Yu Kihara, Anzu Oya, Karen Endo, Rina Oe, Sakura Takeda, Asaki Saso

**Affiliations:** 1 School of Nursing, University of California Los Angeles, Los Angeles, California, USA; 2 Dornogovi Medical School, Mongolian National University of Medical Sciences, Dornogovi, Mongolia; 3 Sho-ei Kai, Social Welfare Corporation, Chiba, Japan; 4 International School of Mongolian Medicine, Mongolian National University of Medical Sciences, Ulaanbaatar, Mongolia; 5 Department of Nursing, Faculty of Human Sciences, Sophia University, Tokyo, Japan

## Abstract

Mongolia, the world’s second-largest landlocked country, has a healthcare system shaped by Soviet and Chinese influences. While its capital, Ulaanbaatar, houses nearly half of the population with well-developed medical facilities, rural and remote areas, including the Gobi region, face significant disparities in healthcare access. Urban migration to Ulaanbaatar is driven by better economic opportunities, healthcare services, and infrastructure. Traditional Mongolian medicine (TMM) remains an integral part of healthcare, particularly in rural areas, where it is often the primary form of treatment. Despite the adoption of universal healthcare coverage, rural healthcare struggles with workforce shortages, outdated infrastructure, and limited resources. Nurses and midwives lack professional autonomy, and preventive care remains underdeveloped. To address these challenges, Mongolia can strengthen global collaborations through its ‘third neighbour policy’, expanding partnerships with countries like the USA and Japan to improve healthcare education and workforce capacity. Enhancing online training, telemedicine, and disease prevention programmes, particularly in rural areas, would further support healthcare development. Expanding nursing and midwifery roles, integrating health screenings into community events, and leveraging digital health technologies can bridge healthcare gaps. A holistic approach integrating modern and traditional medicine can lead to a more resilient, accessible, and culturally appropriate healthcare system.

Mongolia ranks among the 20 largest countries in the world and is the second-largest landlocked country, bordering Russia and China. Over the past century, it has experienced significant influence from both neighbours due to periods of colonisation and geopolitical impact. As a result, its healthcare system has been heavily shaped by the medical practices and structures of China and Russia [1]. Due to its influence, Mongolia modelled its healthcare infrastructure after that of the Soviet Union, with medical professionals trained and operating according to Soviet standards [2]. To date, medical supplies and equipment in Mongolia are largely imported from Russia and China. For instance, during the COVID-19 pandemic, China and Russia supplied the vaccines to Mongolia as well as India, Japan, and the COVAX facility, and there were frequent exchanges of medical training between these two neighbouring countries [3].

Close to half of the Mongolian population is scattered around the country in numerous semi-desert areas called *gobi* and other remote areas, making it the world’s second least densely populated country. Approximately 1.7 million people live in the capital of Ulaanbaatar, with a population density of 704 people per square mile, representing nearly half of the nation’s population [4].

For this reason, a study conducted in 2009 reported significant disparities in the availability of healthcare professionals across Mongolia. In Ulaanbaatar, there were 3.96 physicians per 1000 residents, while this number was substantially lower in the provinces (*aimags*) of Bayankhongor, Khovsgol, Arkhangai, and Bayan-Olgii, which had 1.34, 1.39, 1.45, and 1.47 physicians per 1000 residents, respectively [5]. This urban-rural disparity highlights the significant challenges faced by remote areas. Similarly, in terms of nurses, the highest ratio was observed in the Govisumber province, with 3.69 nurses per 1000 residents, while the lowest was seen in the Ovorkhangai province, with only 2.31 nurses per 1000 residents [5]. This is in contrast to the USA ratio in 2007 when there were 10.6 nurses per 1000 population [6].

**Figure:**
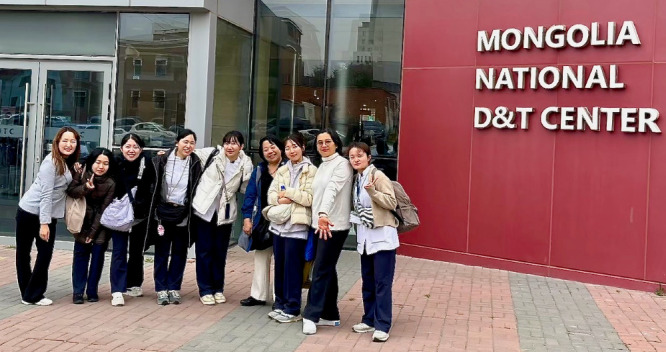
Photo: The 2024 Global Health Practicum Team and authors in front of the Mongolia National Diagnostic and Treatment Center. Source: first author’s personal collection, used with permission of all individuals in the photograph.

The urban migration to Ulaanbaatar, Mongolia’s only metropolitan city, can be attributed to several factors. First, the city offers greater availability of services and better economic opportunities. Residents of Ulaanbaatar have access to a wider range of globally imported goods and increased chances for employment, including positions in multinational institutions [7]. However, this rapid urban expansion has led to significant challenges such as air pollution and traffic congestion. Second, harsh winter conditions have driven generations to relocate to Ulaanbaatar. Many rural areas still lack essential infrastructure, including paved roads, sanitation facilities, gas lines, and electricity. Consequently, younger generations prefer urban living, where these necessities are readily available. Third, the ageing population requires increased healthcare access and resources. In Ulaanbaatar, both traditional Mongolian medicine (TMM) and modern Western treatments are widely accessible and are supported by universal health coverage (UHC), which reduces out-of-pocket transportation costs for Mongolians [8].

After the end of the Cold War with the Soviet Union, Mongolia transitioned into a market economy. As government funding was reallocated, healthcare, education, and social security experienced reduced financial support. This shift in funding priorities has impacted the quality and accessibility of these essential services [9]. Therefore, disparities between the capital city, Ulaanbaatar, and the rest of the country have increased [10]. Here we highlight the challenges and opportunities facing the healthcare system, nursing, and midwifery education in Mongolia, as the country strives to enhance its healthcare system and develop a skilled workforce. We discuss various cultural practices and national policies that improve the country's resilience and position, identify weaknesses in the current environment and policies, and provide future recommendations tailored to existing cultural practices.

## STRENGTHS

There are several effective policies to keep the population young and strong. First, people who meet certain criteria can retire early – for example, women with four or more children or workers in hazardous occupations [11], the majority of whom can meet these requirements. Compared with nearby countries such as Japan, Korea, Taiwan and China, this policy has dramatically increased the size of the labour force, supporting the rest of the population and maintaining a considerable number of young generations [12].

Second, TMM is a vital part of traditional health practices and retains its own distinct and profound theoretical framework of treatment and care. It has been practiced for centuries and is deeply embedded in Mongolia's cultural and religious traditions [13]. Many Mongolians, particularly those in rural and remote areas, rely on TMM to manage their discomfort [14], despite the availability of medical facilities staffed by physicians, nurses, or feldshers in every region.

Additionally, TMM can be more affordable and culturally appropriate, as it aligns closely with local customs and traditions [15]. It is characterised by unique medicinal resources, specialised processing techniques, and particular drug use methods, making it a significant medical tradition with rich cultural and historical roots [16]. In several remote areas, Mongolian nomadic herders rely on TMM for healthcare [17]. This may be due to the healthcare service structure being designed to cover the entire country, yet individuals still need to travel to the nearest clinics or facilities for treatment when experiencing discomfort. The primary healthcare in urban areas is more structured and individuals can be referred to different specialties. Historically, TMM has served as a way to transmit local and herbal knowledge across generations, primarily within families. However, little is known about how ongoing societal transformations may affect the use and transmission of traditional ecological knowledge among herders [18].

TMM has been widely integrated into modernised medical practices in Mongolia. It emphasises the balance between the mind, body, and environment, offering a holistic approach to healthcare. Common treatments include bloodletting, herbal remedies, and acupuncture [19]. Additionally, the co-integration of Easter and Western medicine is prevalent, particularly in the management of symptoms and chronic illnesses. Self-care and symptom management strategies involving the use of herbs in food preparation, such as addressing headaches, diarrhoea, and rashes, are encouraged to reduce discomfort [19]. In rural areas, TMM is more frequently used because of its accessibility and cultural acceptance, as herbal remedies align closely with traditional practices [17]. Moreover, the Ministry of Health in Mongolia supports the integration of TMM by encouraging its practitioners to align their treatments with updated Western medical practices, fostering collaboration between the two systems [20].

Third, Mongolia provides UHC with free access to primary healthcare for all its citizens. A *semashko*-style centralised and hierarchical healthcare system (a single-payer healthcare system and usually funded from the national budget), established during the Soviet era, plays a crucial role in improving the overall health of the population, particularly in rural areas. This single-payer healthcare model ensures that healthcare is free for everyone and is funded by the national budget. The system includes a network of *soum* health centres, which serve as the sole healthcare providers in rural *soums* (the smallest administrative units, similar to counties). These centres offer primary care, whereas referral-level general hospitals at the *aimag* (province) level deliver both primary and secondary healthcare services. However, in many rural regions, particularly in remote areas such as the Gobi Desert, the *aimag*-level hospital is often the only healthcare facility available, serving as a critical lifeline for these communities [21]. Compared with Ulaanbaatar, the capital city, healthcare is provided through polyclinics, district hospitals, tertiary hospitals and specialised centres with plentiful resources from government funding [22].

Thus, these elements reflect the values and strategies that contribute to the unique identity and development of Mongolia’s healthcare.

## WEAKNESSES

Despite positive developments in Mongolia’s healthcare system, several areas still require significant improvement. One substantial challenge is the distance between nomadic regions and provincial hospitals. For instance, the distance from Altanshiree, an area predominantly inhabited by nomadic herders residing in yurts, to Sainshand, the capital of Dornogobi Province, is approximately 94.8 km. This journey can pose substantial barriers to accessing healthcare, especially in emergencies, as it requires navigation of rough terrain largely devoid of road signs and often takes more than two hours. This remoteness can hinder timely medical attention, underscoring the need for improved healthcare access in rural and nomadic areas. For example, healthcare providers in Altanshiree are familiar with the route to Sainshand for provincial care; if more specialised healthcare services are needed, the team of general hospital in Dornogobi provides timely emergency support from the provincial centre. However, emergency crews might need guidance from outside, as most *soum* hospitals are located approximately 95–200 km from Sainshand.

The shortage of healthcare providers in rural areas is also a significant concern. For example, fewer than 10 healthcare professionals serve the entire area at the Altanshiree community hospital. Many of these healthcare workers must visit patients directly in their yurts, providing care in challenging and often isolated conditions. This limited workforce strains the system and hampers the ability to offer timely and comprehensive medical services in rural communities. Other challenges such as outdated infrastructure and limited medical resources hinder the system's ability to fully meet the population's needs. Addressing these gaps is critical to advancing healthcare services and ensuring equitable access for all Mongolians [21].

Furthermore, the lack of professional recognition and autonomy for nurses and midwives in Mongolia is a growing concern for population health, especially with half of the population residing in rural areas where healthcare providers, including nurses, are scarce [23,24]. Nurses in Mongolia are still largely viewed as assistants to doctors and are unable to act independently in patient and perinatal care without a doctor’s orders. Furthermore, most nursing and midwifery programmes are provided in medical schools and are taught by physician-trained faculty and clinical nurses, with few highly-educated nurses serving as faculties and/or heads of nursing departments in nursing schools [23,24]. Medical schools in rural areas have diverse levels of experience in training nurses and midwives to serve local communities. Some schools offer three-year programmes, while others provide four-year education for nurses and midwives. Therefore, multilevel investments in both nursing educators and clinical practitioners are crucial to strengthening the roles of nurses and midwives at the national level, ensuring consistent quality and competency across different schools. This will help ensure the delivery of high-quality midwifery and nursing care, particularly in underserved rural areas, and promote a more autonomous role for these professionals in healthcare delivery.

Additionally, there is a lack of disease prevention concepts and practices in Mongolia. Currently, the leading causes of death are diseases of the respiratory, digestive, genitourinary, and circulatory systems [9]. Others have reported that heart disease, trauma, cancer, and poisonings have resulted in most years of life being lost in Mongolia [21]. Approximately half of the causes of out-of-hospital deaths can be treated by emergency/critical care interventions [22]. In Mongolian culture, food serves as a vital source of comfort for individuals enduring harsh weather conditions. Salty milk tea, sweet desserts, and heavy mutton and beef cuisines are popular foods in the country. Despite this, hypertension, diabetes and colon cancer screenings are not routinely provided as part of the national guidelines for health screenings, although the Ministry of Health in Mongolia conducted a health screening initiative across four rural regions and the capital city in 2022 and 2023 [25].

Additionally, disparities in funding and resources between rural and urban areas need to be addressed. Although UHC is funded by the government, accessibility remains a challenge in rural regions. Compared to urban areas with better infrastructure and services, *soum* health centres are often located in remote areas, where residents face greater difficulties accessing healthcare due to limited resources such as family support and transportation [26]. Moreover, elderly individuals in rural areas face significant challenges in accessing the medical care they need [8].

## RECOMMENDATIONS

Global institutions can help advance healthcare in Mongolia. First, Mongolia’s ‘third neighbors’, such as India, Japan, South Korea, and the United States, can help reduce its reliance on China and Russia by fostering direct engagement with other nations. Agencies from these foreign countries can support Mongolia in adopting updated WHO guidelines on disease prevention [1]. Nursing professionals from countries such as the United States and Japan, with their advanced knowledge and practices, could explore opportunities to invest in Mongolia's healthcare workforce. By sharing expertise in health promotion and disease prevention, these partnerships could significantly enhance Mongolia's public health capacity and improve overall healthcare outcomes. To date, the ‘third neighbour policy’ has contributed to promoting international collaboration [27], improving healthcare infrastructure [28], and increasing access to medical resources and training [24,29]. While the original purpose of the policy was for geopolitical situations, it has had positive and indirect impacts on the health sector, particularly in terms of modernisation, rural healthcare access, and global health partnerships.

Second, strengthening online training infrastructure to connect the capital city with *gobi* regions is essential. As the government invests heavily in Ulaanbaatar, updated technology and medical knowledge can be effectively shared through online platforms. This will enhance telemedicine for residents in remote areas who have access to proper equipment. Additionally, integrating updated technology will ensure that nursing and midwifery education aligns with current best practices globally. Expanding global partnerships with international nursing and midwifery educators can further enrich education and training, fostering a more comprehensive and globally informed healthcare workforce. Presently, several nursing institutions in Japan contribute significantly to continuing education for nurses, both within hospitals and nursing schools, fostering professional development and improving healthcare delivery [27,29].

Third, expanding the scope of nursing roles to include primary care, disease prevention, and health promotion is crucial. Given that half of Mongolia's population is dispersed across rural areas, nurses can play a vital role in conducting basic symptom screenings and referring severe cases to *soum* hospitals. This approach would optimise financial and human resources, making healthcare delivery more feasible and effective in the *gobi* regions. For midwives, several comprehensive policy recommendations have been proposed, including the creation of a chief midwife management position, expanding the role of midwives, and developing postgraduate midwifery programmes and training to increase the number of skilled healthcare providers specialising in women’s and children’s health [23].

Finally, health screenings for residents can be expanded by integrating them into community and religious events, making health campaigns more accessible and culturally relevant. Additionally, leveraging digital health technologies can improve healthcare access in remote locations. Mobile clinics offer a practical solution to reach residents in underserved areas. Furthermore, partnering with international non-governmental organisations can facilitate the delivery of basic disease prevention strategies through local collaborations, ensuring that interventions are tailored to the community's needs.

Our goal with this viewpoint is to facilitate the discussion about potentially expanding nursing roles, connecting with domestic and global collaborators to increase the uptake of current healthcare knowledge, and integrating TMM into local practices, while providing care in remote areas. These strategies could lead to a more robust, accessible, and culturally sensitive healthcare system, improving the well-being of individuals and communities across Mongolia.
